# Development of Guanidine-Bisurea Bifunctional Organocatalysts with a Chiral Pyrrolidine Moiety and Application to α-Hydroxylation of Tetralone-Derived β-Keto Esters

**DOI:** 10.3390/molecules200712590

**Published:** 2015-07-10

**Authors:** Minami Odagi, Kan Takayama, Makoto Sato, Masahiro Yamanaka, Kazuo Nagasawa

**Affiliations:** 1 Department of Biotechnology and Life Science, Tokyo University of Agriculture and Technology, 2-24-16, Naka-cho, Koganei city, 184-8588 Tokyo, Japan; E-Mails: odagi@cc.tuat.ac.jp (M.O.); k-takayama@koeichem.co.jp (K.T.); 2 Department of Chemistry, Faculty of Science, Rikkyo University, 3-34-1, Nishi-Ikebukuro, Toshima-ku, 171-8501 Tokyo, Japan; E-Mails: msato_chem@rikkyo.ac.jp (M.S.); myamanak@rikkyo.ac.jp (M.Y.)

**Keywords:** guanidine, urea, organocatalyst, α-hydroxylation

## Abstract

Novel guanidine-bisurea bifunctional organocatalysts **5** bearing a chiral pyrrolidine moiety on guanidine were designed with the guidance of DFT calculations. The resulting organocatalysts **5** were examined for α-hydroxylation of tetralone-derived β-keto esters, and good selectivity was obtained with **5j** bearing a methoxymethyl ether-containing chiral pyrrolidine moiety.

## 1. Introduction

**Figure 1:**
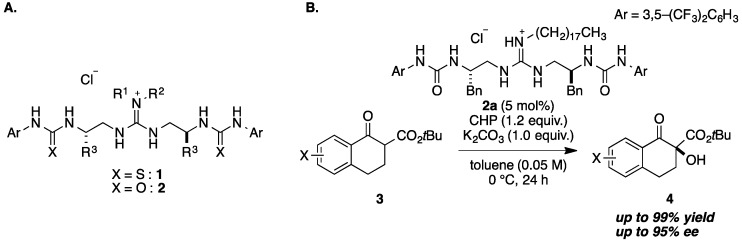
(**A**) Structures of guanidine-bis(thio)urea bifunctional organocatalysts **1** and **2**; (**B**) α-Hydroxylation of tetralone-derived β-keto ester **3** using **2a**.

We have reported a series of structurally flexible guanidine-bis(thio)urea organocatalysts **1** and **2** for asymmetric reactions (Figure 1A) [1,2], including Henry reactions [3], Mannich-type reactions [4,5], Michael reactions [6,7], and Friedel-Crafts reactions [8,9]. In these reactions, guanidine and (thio)urea groups in the catalysts are proposed to interact with nucleophiles and electrophiles, respectively, and new bond-forming processes proceed efficiently due to synergistic proximity. Moreover, chiral spacers connecting the guanidine and (thio)urea groups in the catalysts serve to construct a chiral environment for the reaction transition state, enabling highly enantioselective reactions to occur.

**Figure 2:**
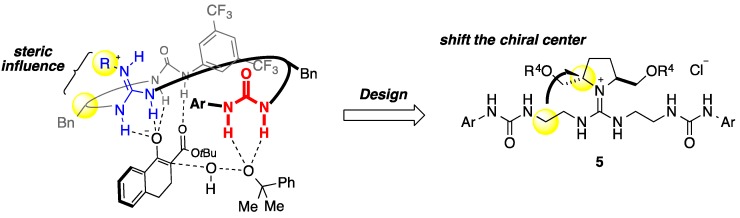
Design of our chiral pyrrolidine-derived guanidine-bisurea bifunctional organocatalysts **5**.

We have recently developed an enantioselective α-hydroxylation of tetralone-derived β-keto esters **3** by utilizing guanidine-urea catalyst **2a** in the presence of cumene hydroperoxide (CHP), affording the α-hydroxylation products **4** in high yield with high enantioselectivity (Figure 1B) [10]. We considered that guanidine interacts with β-keto ester, and the urea group activates CHP, and we performed DFT calculations to examine the feasibility of this idea [11]. The calculations indicated that enolate interacts with functional groups of guanidine and urea in the catalyst, and the remainder of the urea group activates the oxidant, CHP. Moreover, steric interactions between the R group of guanidine and the substituent of the Bn group in the chiral spacer were revealed to contribute to construction of the chiral reaction environment (Figure 2). Based on the calculated transition state model, we focused on shifting the chiral site in the catalyst from the spacer to the substituent on guanidine. By shifting the chiral site, an alternative control of the chiral environment was expected, and we designed compounds **5** as a novel type of guanidine-bisurea bifunctional catalysts (Figure 2) [12]. In the previous catalyst **2a**, chiral spacers were synthesized from the corresponding amino acids, and their diversity was limited. On the other hand, various substituents can be installed in the R^4^ group at the optically active pyrrolidine moiety in compound **5**, so it should be possible to construct a range of chiral environments. In this paper, we describe the synthesis of a new type of guanidine-bisurea bifunctional catalysts **5**, as well as their application to α-hydroxylation of tetralone-derived β-keto esters **3a** [13,14,15,16,17].

## 2. Results and Discussion

**Scheme 1:**
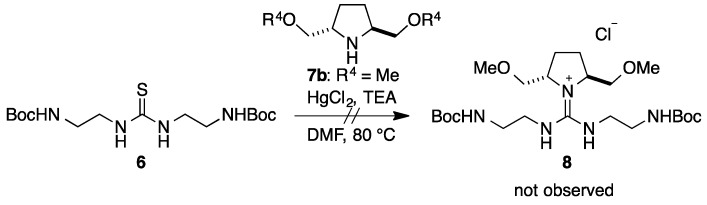
Failure to obtain guanidine **8** from **6** and **7b**.

Using the synthetic route established for **1** and **2**, we examined the introduction of a chiral pyrrolidine **7b** [18] into thiourea **6** to generate chiral guanidine **8** (Scheme 1). However, the reaction did not proceed under various conditions. Thus, we concluded that the reactivity of chiral pyrrolidine **7b** may be too low owing to steric hindrance of the 2,5-substituents.

**Scheme 2:**
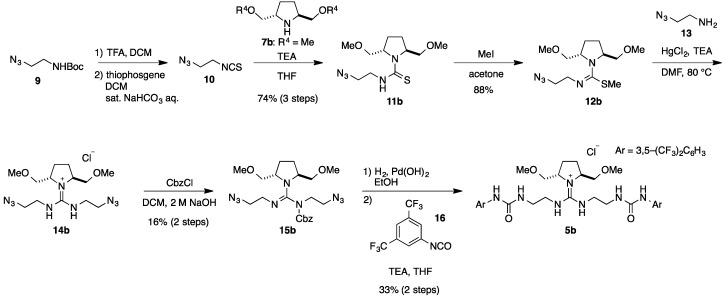
Synthesis of chiral pyrrolidine-derived guanidine-bisurea bifunctional organocatalysts **5b**.

**Scheme 3:**
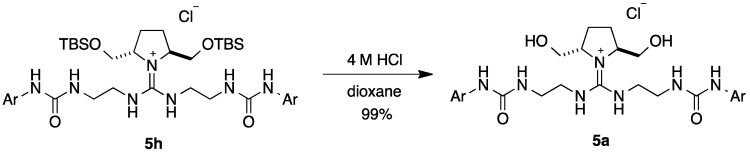
Synthesis of chiral pyrrolidine-derived guanidine-bisurea bifunctional organocatalyst **5a**.

Then, we changed the introduction order of pyrrolidine **7** (Scheme 2). Thus, reaction of pyrrolidine **7b** with isothiocyanate **10**, obtained from NHBoc azide **9**, in the presence of mercury (II) chloride gave corresponding thiourea **11b** in 74% from **9**. After conversion of the thiourea **11b** into methyl pseudo-thiourea **12b** by treatment with methyl iodide, the resulting **12b** was reacted with amine **13** in the presence of mercury (II) chloride and triethylamine to give guanidinium salt **14b**, which was further reacted with benzyloxycarbonyl chloride in the presence of triethylamine to give Cbz-protected guanidine **15b** in 16% yields. Then, reduction of azide and deprotection of Cbz group took place simultaneously by hydrogenolysis in the presence of Pd(OH)_2_, and resulting diamine was reacted with 3,5-bis(trifuluoromethyl)-phenyl isocyanate **16** to give guanidine-bisurea **5b** in 33% from **15b**. Based upon the present route (Scheme 2), **5c**–**j** (R = *n*-Pr, *n*-Decyl, Bn, CH_2_(1-Naphthyl), CH_2_(2-Naphthyl), TBS and TIPS) were obtained by changing the R^4^ group on the chiral pyrrolidine, respectively. Furthermore, guanidine-bisurea catalyst **5a**, which has hydroxyl group on the chital pyrrolidine, was synthesized from catalyst **5h** by deprotection of TBS ether with hydrochloric acid (Scheme 3).

**Figure 3:**
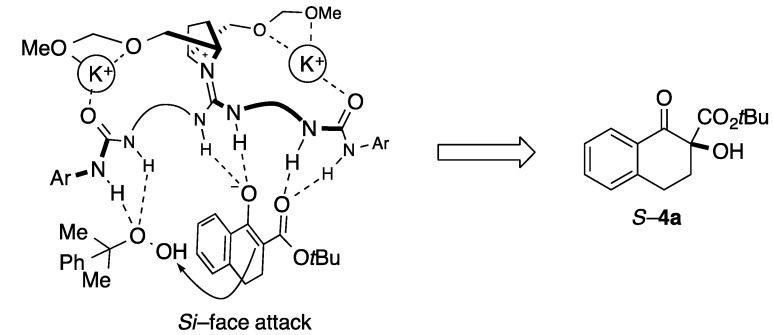
Plausible transition state model of α-hydroxylation of **3a** in the presence of **5j**.

With the novel guanidine-bisurea catalysts **5a**–**j** in hand, α-hydroxylation of tetralone-derived keto ester **3a** was examined (Table 1). The reaction was carried out by following conditions, *i.e.*, CHP (1.2 equiv.), K_2_CO_3_ (1 equiv.) in toluene at 0 °C, which we previously reported as optimized conditions in the presence of catalyst **2a**. In case of **5a**, catalytic activity was low, and hydroxylation product **4a** was only obtained in 5% with 30% *ee* (entry 1). Then, **5b**–**d** with alkyl ether groups of methyl, *n*-propyl, and *n*-decyl ethers were examined. In these cases, reactivities were increased, and moderate yield of **4a** was obtained in 49%, 49% and 47%, respectively. Moreover, moderate enantioselectivities (38%–50% *ee*) were obtained (entries 2–4). Then, benzyl, 1-naphtylmethyl, and 2-naphtylmethyl ethers of **5e**–**g** were explored as catalyst with expecting more effective interactions between oxidant of CHP with catalyst through the π-π interactions (entries 5–7). Unfortunately, however, reactivities and selectivities were still moderate, and hydroxylated **4a** was obtained with 36%, 58%, 55% yield in 30%, 40%, and 32% *ee*, respectively. In case of more bulky substituents of silyl ethers of TBS **5h** and TIPS **5i**, reactivity was slightly increased, however, selectivity was still moderate (entries 8 and 9). On the other hand, compound **5j** which has methoxymethyl (MOM) ether group catalyzed the reaction efficiently, and hydroxylated **4a** was obtained with 73% yield in 65% *ee*, which were the best results among the catalysts we examined. Since the MOM group has two oxygen atoms, we currently proposed that those two oxygen atoms might considerably contribute the construction of chiral reaction environment through the chelations of multidentate nature of potassium with β-keto ester (Figure 3) [19,20]. Actually, by changing the base from potassium carbonate to cesium carbonate, which has bigger size of cation radius than potassium, enantioselectivity was dropped to 48% *ee* without affecting the reactivity (73% yield, Table 1, entry 11). Further improvements of the selectivity for the reaction by utilizing new type of catalysts **5** are in progress based upon the present findings.

**Table 1:** Investigation of α-hydroxylation of **3a** using **5**^a^. 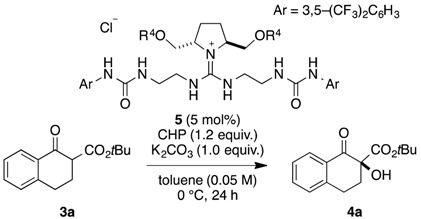

Entry	5		4a	
		R^4^	Yield [%] ^b^	*ee* [%] ^c^
1	**5a**	H	5	30
2	**5b**	Me	49	38
3	**5c**	*n-*Pr	49	50
4	**5d**	*n-*Decyl	47	47
5	**5e**	Bn	36	30
6	**5f**	CH_2_(1-Naphtyl)	58	40
7	**5g**	CH_2_(2-Naphtyl)	55	32
8	**5h**	TBS	55	49
9	**5i**	TIPS	63	34
10	**5j**	MOM	73	65
11 ^d^	**5j**	MOM	73	48

^a^ Reaction conditions: **3a** (0.1 mmol), CHP (0.12 mmol) and K_2_CO_3_ (0.1 mmol) in the presence of **5** (5 mol %) in toluene (2.0 mL) at 0 °C for 24 h. ^b^ Isolated yield. ^c^ Determined by HPLC analysis using a chiral stationary phase. ^d^ Cs_2_CO_3_ (0.1 mmol) was used instead of K_2_CO_3_ as a base.

## 3. Experimental Section

### 3.1. General Remarks

Flash chromatography was performed using silica gel 60 (spherical, particle size 0.040–0.100 mm. Kanto Co., Tokyo, Japan). Optical rotations were measured on a JASCO P-2200 polarimeter (JASCO, Tokyo, Japan). ^1^H- and ^13^C-NMR spectra were recorded on AL300 (JEOL), ECX400 (JEOL), or AVANCE400 (Bruker) instruments (JEOL, Tokyo, Japan). Chemical shifts in [*d*_4_]MeOH was reported in the scale relative to [*d*_4_]MeOH (δ = 3.30 ppm) for ^1^H-NMR spectroscopy. For ^13^C-NMR spectra, chemical shift was reported in the scale relative to [*d*_4_]MeOH (δ = 49.0 ppm) (internal reference). Mass spectra were recorded on a JMS-T100LC (JEOL) spectrometer (JEOL). HPLC analysis on a chiral stationary phase was performed on JASCO 800-series instruments (JASCO). A Daicel Chiralpak AD-H column (0.46 cm × 25 cm) was used with hexane/ethanol as the eluent.

### 3.2. Typical Procedure for α-Hydroxylation of β-Keto Ester **3a** Using **5**

A test tube equipped with a magnetic stirring bar was charged with catalyst **5** (0.005 mmol), tetralone-derived β-keto ester **3a** (0.1 mmol), K_2_CO_3_ (0.1 mmol), and toluene (2.0 mL) at room temperature. The mixture was cooled to 0 °C and stirred for 10 min. Then, cumene hydroperoxide (0.12 mmol) was added, and stirring was continued at 0 °C for 24 h. The reaction mixture was directly purified by column chromatography on silica gel (*n*-hexane/ethyl acetate 50:1 to 20:1) to give the product **4a**. Enantiomeric excess and absolute configuration were determined by HPLC analysis of the product on a chiral column (DAICEL Chiralpak AD-H) with *n*-hexane/2-propanol (95:5).

### 3.3. Characterizations of Novel Guanidine-Bisurea Bifunctional Organocatalysts **5a**–**j**

**5a**: ${\left[\alpha \right]}_{D}^{25}$ = +41.6 (*c* 2.1, MeOH); ^1^H-NMR (300 MHz, CD_3_OD) δ 7.98 (s, 4H), 7.43 (s, 2H), 4.13 (brs, 2H), 3.61 (dd, *J* = 11.0 Hz, 2H), 3.52–3.37 (m, 10H) 3.33–3.24 (m, 2H), 2.22–2.02 (m, 2H), 1.95–1.77 (m, 2H); ^13^C-NMR (75 MHz, CD_3_OD) δ 159.8, 158.8, 144.0, 133.9 (q, *J*_C-F_ = 32.6 Hz), 125.6 (q, *J*_C-F_ = 271.3 Hz), 119.9, 116.5, 64.4, 64.1, 46.7, 41.1, 28.3 ; HRMS (ESI, M-Cl) calcd for C_29_H_32_F_12_N_7_O_4_ 770.2324, found 770.2364.

**5b**: ${\left[\alpha \right]}_{D}^{25}$ = +38.3 (*c* 1.0, CHCl_3_); ^1^H-NMR (300 MHz, CD_3_OD) δ 7.99 (s, 4H), 7.43 (s, 2H), 4.21 (brs, 2H), 3.53–3.24 (m, 12H), 3.19 (s, 6H), 2.22–2.03 (m, 2H), 1.87–1.69 (m, 2H); ^13^C-NMR (75 MHz, CD_3_OD) δ 160.2, 158.7, 144.1, 133.9 (q, *J*_C-F_ = 33.2 Hz), 125.6 (q, *J*_C-F_ = 272.4 Hz), 119.8, 116.5, 75.5, 62.1, 60.3, 46.7, 40.9, 28.6; HRMS (ESI, M-Cl) calcd for C_31_H_36_F_12_N_7_O_4_ 798.2637, found 798.2594.

**5c**: ${\left[\alpha \right]}_{D}^{25}$ = +49.2 (*c* 1.0, CHCl_3_); ^1^H-NMR (300 MHz, CD_3_OD) δ 8.01 (s, 4H), 7.46 (s, 2H), 4.24 (brs, 2H), 3.53–3.12 (m, 16H), 2.28–2.08 (m, 2H), 1.94–1.76 (m, 2H), 1.52–1.37 (m, 4H), 0.78 (t, *J* = 7.2 Hz, 6H); ^13^C-NMR (75 MHz, CD_3_OD) δ 159.9, 158.8, 144.1, 134.0 (q, *J*_C-F_ = 32.6 Hz), 125.6 (q, *J*_C-F_ = 271.3 Hz), 119.8, 116.5, 75.1, 73.4, 62.3, 46.9, 41.0, 28.9, 24.7, 11.7; HRMS (ESI, M-Cl) calcd for C_35_H_44_F_12_N_7_O_4_ 854.3263, found 854.3236.

**5d**: ${\left[\alpha \right]}_{D}^{25}$ = +30.4 (*c* 1.0, CHCl_3_); ^1^H-NMR (300 MHz, CD_3_OD) δ 8.02 (s, 4H), 7.44 (s, 2H), 4.23 (brs, 2H), 3.51–3.23 (m, 16H), 2.25–2.06 (m, 2H), 1.89–1.74 (m, 2H), 1.48–1.31 (m, 4H), 1.30–1.05 (m, 28H), 0.82 (t, *J* = 6.5 Hz, 6H); ^13^C-NMR (75 MHz, CD_3_OD) δ 159.9, 158.7, 144.1, 134.0 (q, *J*_C-F_ = 33.2 Hz) 125.6 (q, *J*_C-F_ = 272.4 Hz), 119.8, 116.5, 73.5, 62.3, 46.9, 41.0, 33.9, 31.6, 31.4, 31.3, 28.9, 28.0, 24.6, 15.3; HRMS (ESI, M-Cl) calcd for C_49_H_72_F_12_N_7_O_4_ 1050.5454, found 1050.5491.

**5e**: ${\left[\alpha \right]}_{D}^{25}$ = +21.5 (*c* 1.0, CHCl_3_); ^1^H-NMR (300 MHz, CD_3_OD) δ 7.97 (s, 4H), 7.44 (s, 2H), 7.28–7.11 (m, 10H), 4.39 (s, 4H), 4.29 (brs, 2H), 3.52 (dd, *J* = 9.6 Hz, 2H), 3.45–3.17 (m, 10H), 2.28–2.10 (m, 2H), 1.91–1.78 (m, 2H); ^13^C-NMR (75 MHz, CD_3_OD) δ 159.8, 158.8, 144.0, 140.0, 133.9 (q, *J*_C-F_ = 33.2 Hz), 130.3, 129.8, 129.7, 125.6 (q, *J*_C-F_ = 272.4 Hz), 119.8, 116.5, 75.2, 72.7, 62.1, 46.9, 40.9, 28.8; HRMS (ESI, M-Cl) calcd for C_43_H_44_F_12_N_7_O_4_ 950.3263, found 950.3240.

**5f**: ${\left[\alpha \right]}_{D}^{25}$ = −3.8 (*c* 1.4, CHCl_3_); ^1^H-NMR (400 MHz, CD_3_OD) δ 7.92 (s, 4H), 7.77–7.62 (m, 8H), 7.41 (s, 2H), 7.38–7.27 (m, 6H), 4.54 (s, 4H), 4.33 (brs, 2H), 3.58 (dd, *J* = 10.1 Hz, 2H), 3.45 (dd, *J* = 9.6 Hz, 2H), 3.37–3.12 (m, 8H), 2.34–2.11 (m, 2H), 1.97–1.80 (m, 2H); ^13^C-NMR (100 MHz, CD_3_OD) δ 159.8, 158.7, 143.9, 137.4, 135.4, 135.3, 133.9 (q, *J*_C-F_ = 33.6 Hz), 130.1, 129.7, 129.5, 128.7, 128.1, 127.9, 127.7, 125.6 (q, *J*_C-F_ = 272.2 Hz), 119.9, 116.6, 75.4, 72.8, 62.2, 46.8, 40.9, 28.6; HRMS (ESI, M-Cl) calcd for C_49_H_44_F_12_N_7_O_4_ 1050.3576, found 1050.3596.

**5g**: ${\left[\alpha \right]}_{D}^{25}$ = −7.7 (*c* 1.0, CHCl_3_); ^1^H-NMR (300 MHz, CD_3_OD) δ 7.93 (s, 4H), 7.75–7.63 (m, 8H), 7.42 (s, 2H), 7.38–7.30 (m, 6H), 4.57 (s, 4H), 4.34 (brs, 2H), 3.60 (dd, *J* = 10.0 Hz, 2H), 3.47 (dd, *J* = 10.0 Hz, 2H), 3.37–3.15 (m, 8H), 2.31–2.15 (m, 2H), 2.01–1.84 (m, 2H); ^13^C-NMR (100 MHz, CD_3_OD) δ 159.7, 158.7, 143.9, 137.4, 135.4, 135.2, 133.9 (q, *J*_C-F_ = 32.6 Hz), 130.1, 129.7, 129.5, 128.6, 128.0, 127.9, 127.7, 125.6 (q, *J*_C-F_ = 272.2 Hz), 119.8, 116.5, 75.3, 72.8, 62.2, 46.8, 40.8, 28.8; HRMS (ESI, M-Cl) calcd for C_49_H_44_F_12_N_7_O_4_ 1050.3576, found 1050.3529.

**5h**: ${\left[\alpha \right]}_{D}^{25}$ = −25.6 (*c* 1.0, CHCl_3_); ^1^H-NMR (400 MHz, CD_3_OD) δ 8.04 (s, 4H), 7.46 (s, 2H), 4.19 (brs, 2H), 3.70 (dd, *J* = 10.5 Hz, 2H), 3.61–3.38 (m, 10H), 2.25–2.10 (m, 2H), 1.97–1.85 (m, 2H), 0.79 (s, 18H), −0.03 (s, 6H), −0.04 (s, 6H); ^13^C-NMR (100 MHz, CD_3_OD) δ 159.2, 158.8, 144.1, 134.0 (q, *J*_C-F_ = 33.6 Hz), 125.6 (q, *J*_C-F_ = 272.2 Hz), 119.9, 116.6, 65.0, 64.0, 47.6, 41.0, 28.8, 27.1, 19.8, −4.57, −4.65; HRMS (ESI, M-Cl) calcd for C_41_H_60_F_12_N_7_O_4_Si_2_ 998.4054, found 998.4007.

**5i**: ${\left[\alpha \right]}_{D}^{25}$ = −30.7 (*c* 1.0, CHCl_3_); ^1^H-NMR (300 MHz, CD_3_OD) δ 8.04 (s, 4H), 7.50 (s, 2H), 4.23 (brs, 2H), 3.83 (dd, *J* = 10.7 Hz, 2H), 3.63 (dd, *J* = 10.7 Hz, 2H), 3.56–3.36 (m, 8H), 2.33–2.15 (m, 2H), 2.11–1.92 (m, 2H), 1.11–0.93 (m, 42H); ^13^C-NMR (100 MHz, CD_3_OD) δ 159.5, 158.4, 144.1, 134.1 (q, *J*_C-F_ = 32.6 Hz), 125.7 (q, *J*_C-F_ = 271.3 Hz), 119.8, 116.7, 65.1, 64.2, 48.3, 40.9, 28.8, 19.3, 13.9; HRMS (ESI, M-Cl) calcd for C_47_H_72_F_12_N_7_O_4_Si_2_ 1082.4993, found 1082.4969.

**5j**: ${\left[\alpha \right]}_{D}^{25}$ = +16.8 (*c* 1.0, CHCl_3_); ^1^H-NMR (400 MHz, CD_3_OD) δ 8.02 (s, 4H), 7.46 (s, 2H), 4.51 (s, 4H), 4.31 (brs, 2H), 3.57 (m, *J* = 10.5 Hz, 2H), 3.53–3.43 (m, 10H), 3.24 (s, 6H), 2.30–2.17 (m, 2H), 1.98–1.84 (m, 2H); ^13^C-NMR (100 MHz, CD_3_OD) δ 159.7, 158.8, 144.0, 133.9 (q, *J*_C-F_ = 32.6 Hz), 127.0 (q, *J*_C-F_ = 272.2 Hz), 119.9, 116.5, 98.6, 70.0, 62.1, 56.6, 46.9, 40.1, 28.7 ; HRMS (ESI, M-Cl) calcd for C_33_H_40_F_12_N_7_O_6_ 858.2848, found 858.2828.

^1^H- and ^13^C-NMR date for chiral pyrrolidine-derived guanidine-bisurea bifunctional organocatalysts **5a**–**j** were in the supplementary material.

## 4. Conclusions

In summary, we have designed novel guanidine-bisurea bifunctional organocatalysts **5**, which have chiral substituents on the pyrrolidine ring, based on the DFT calculation experiments. Synthesis of compound **5** was accomplished by changing the installation order of amines into isothiocyanate **11**. Then, catalytic activity of the newly designed **5** was examined by enantioselective α-hydroxylation of β-keto ester **3a**. In this reaction, **5j** bearing MOM group on the chiral pyrrolidine moiety was effective, and hydroxylated product of **3a** was obtained with 73% yield in 65% *ee*. We currently believe that two oxygen atoms in MOM group considerably contribute the regulation of transition state to construct chiral reaction environment through the chelations of potassium with β-keto esters. Further investigations about the transition state of this catalytic reaction are under way in our laboratory.

## Supplementary Materials

Supplementary materials of spectral data for **5a**–**j** can be accessed at: http://www.mdpi.com/1420-3049/20/07/12590/s1.
