# Supplementary material for: Development of Guanidine-Bisurea Bifunctional Organocatalysts with a Chiral Pyrrolidine Moiety and Application to α-Hydroxylation of Tetralone-Derived β-Keto Esters
Source: Molecules. 2015 Jul 10;20(7):12590–8. doi: 10.3390/molecules200712590 (PMC6331824; doi:10.3390/molecules200712590)
Supplement: Supplementary file 1 [file molecules-20-12590-s001.pdf]

Supplementary Materials

^{1}H- and ^{13}C-NMR date for chiral pyrrolidine-derived guanidine-bisurea bifunctional organocatalysts 5a–j

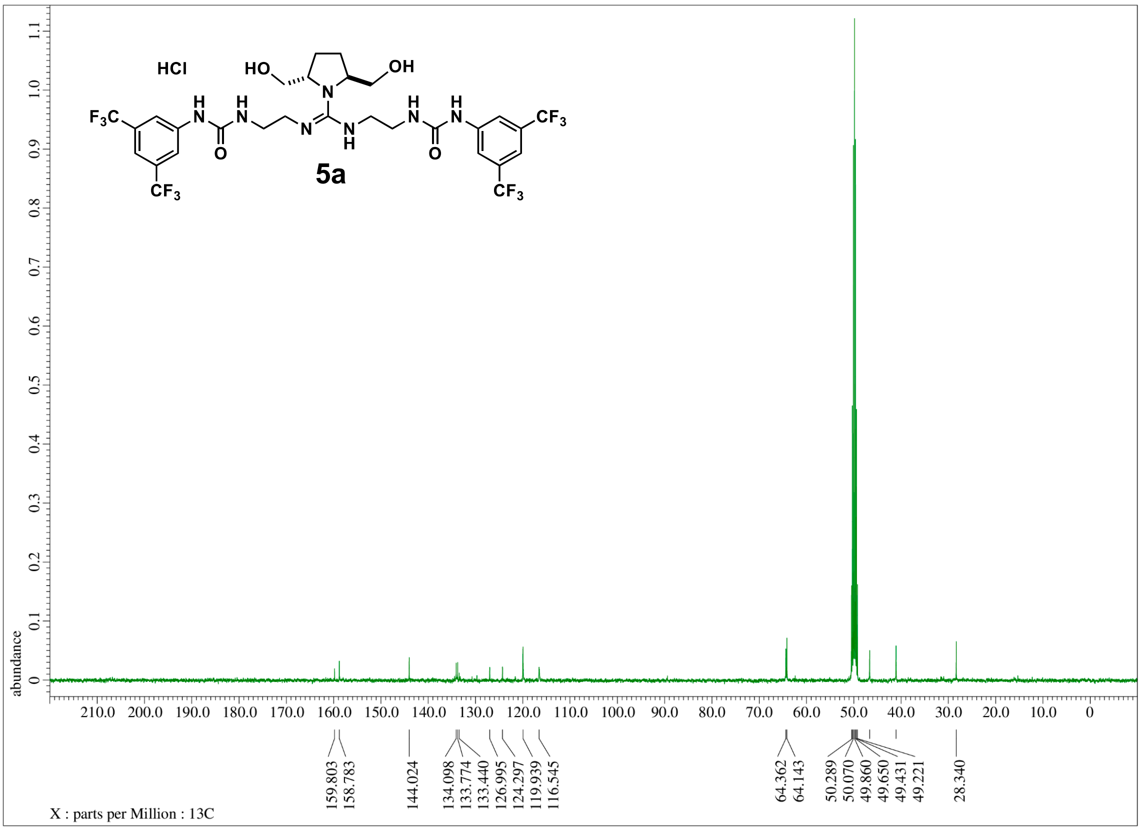

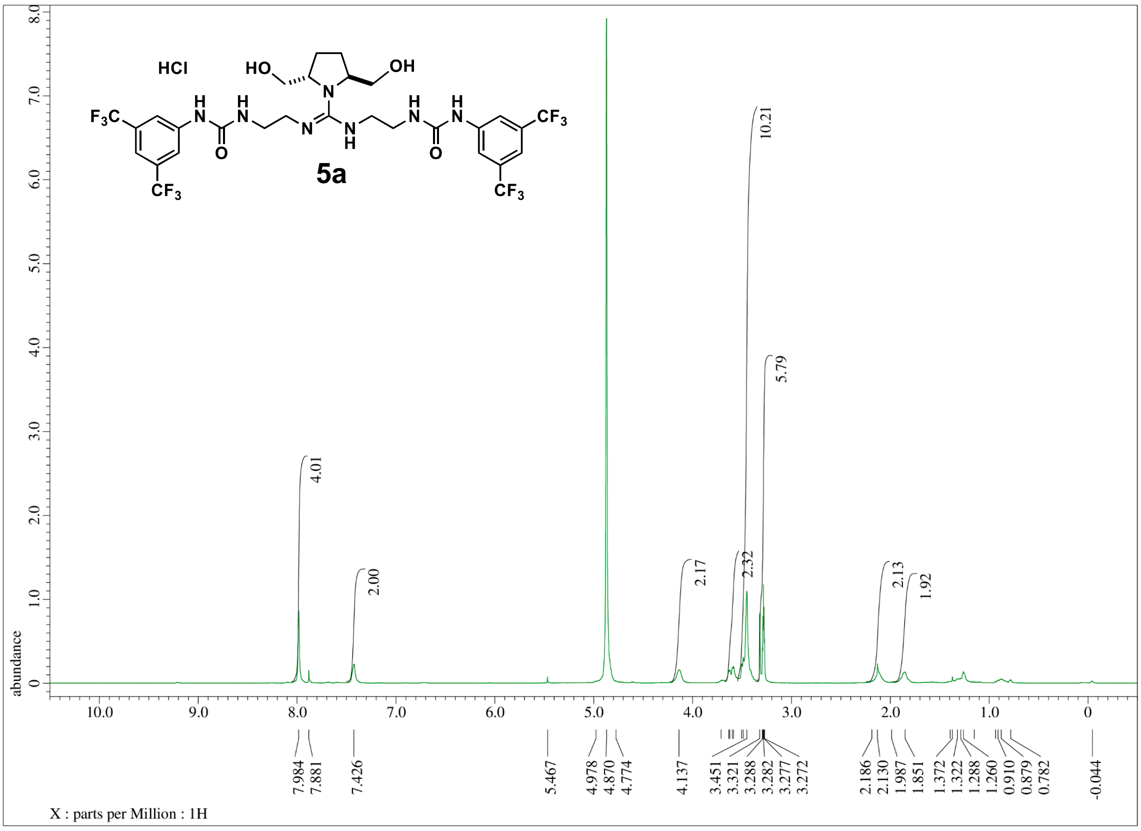

Figure S1. Copies of NMR spectra of **5a**.

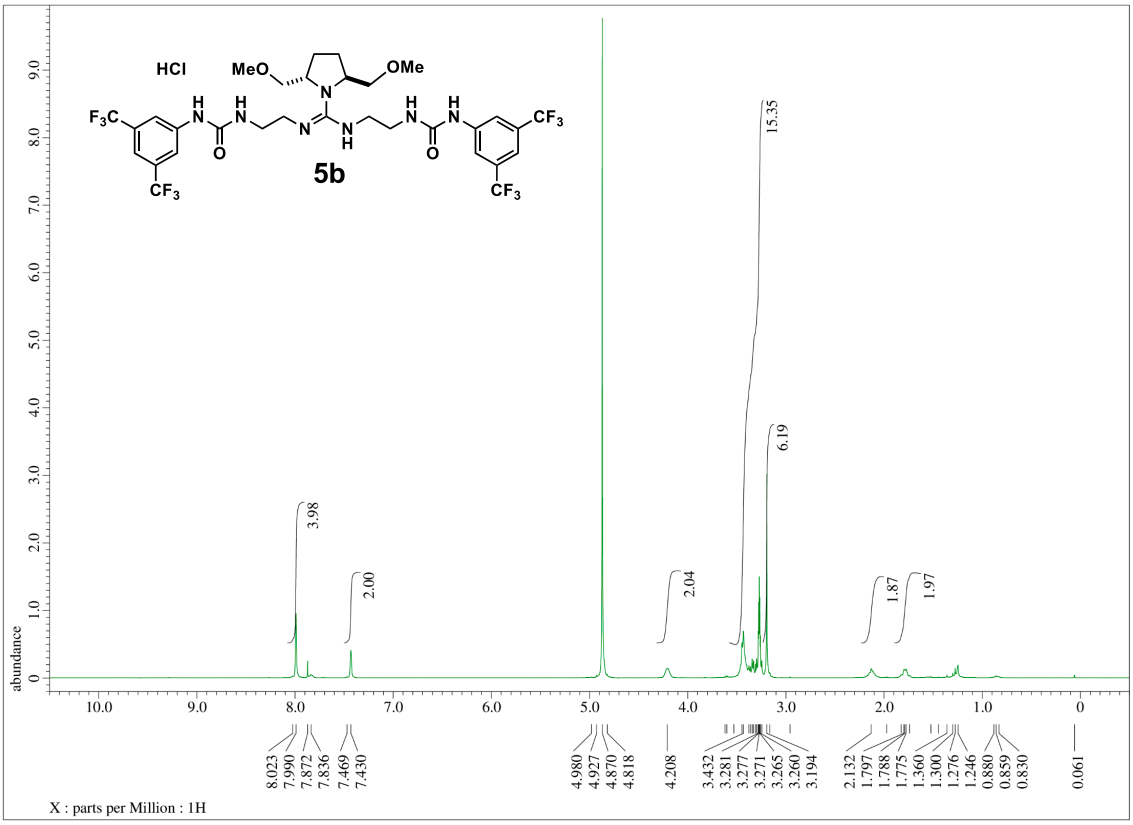

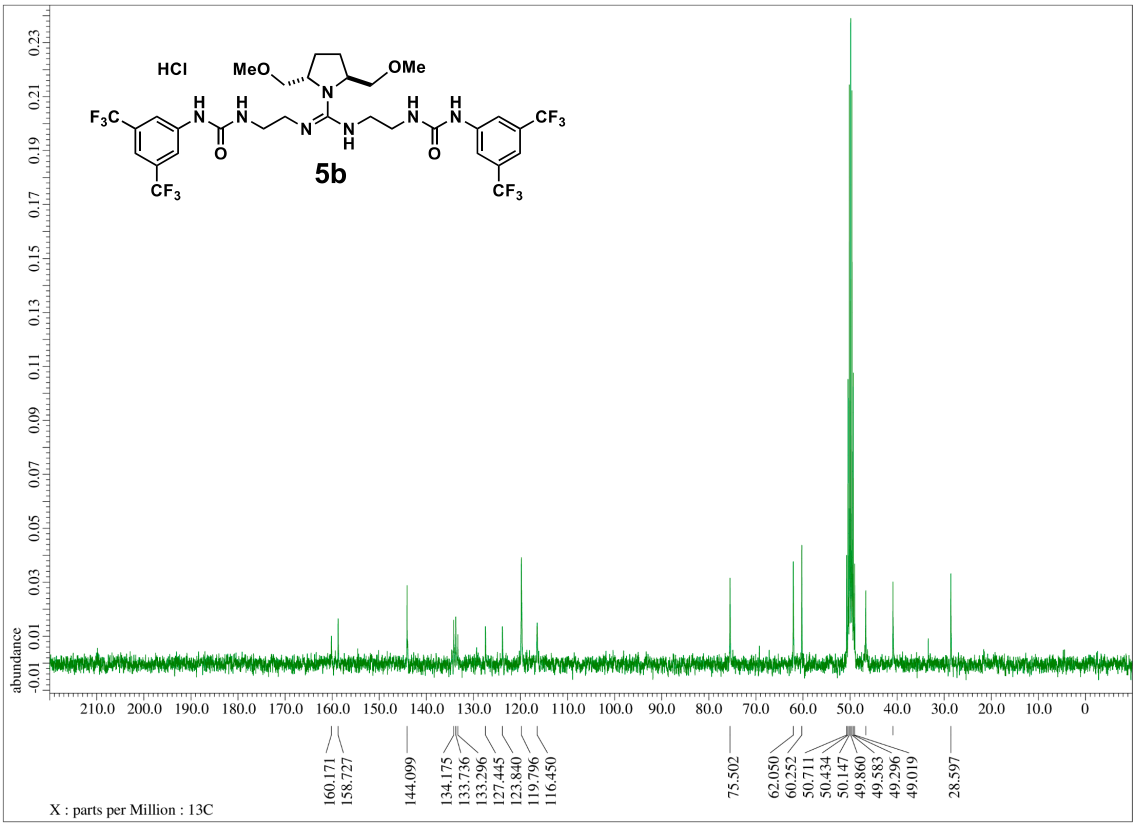

Figure S2. Copies of NMR spectra of **5b**.

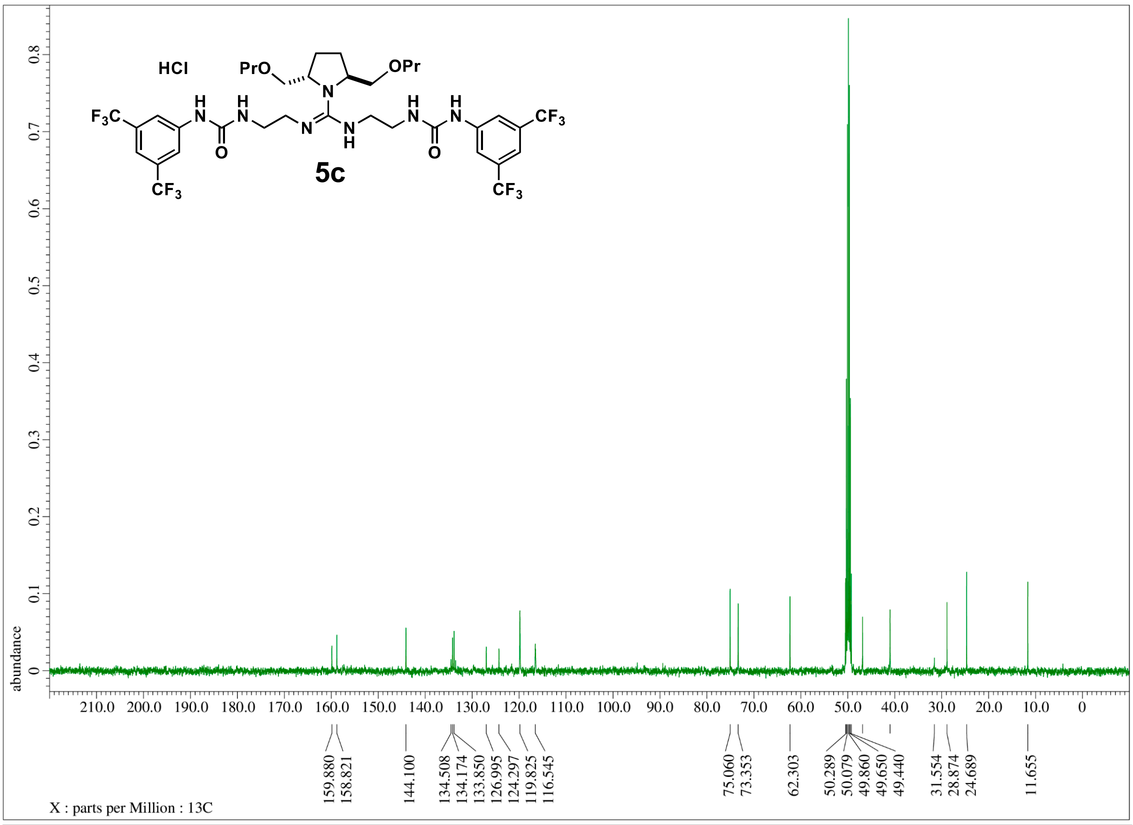

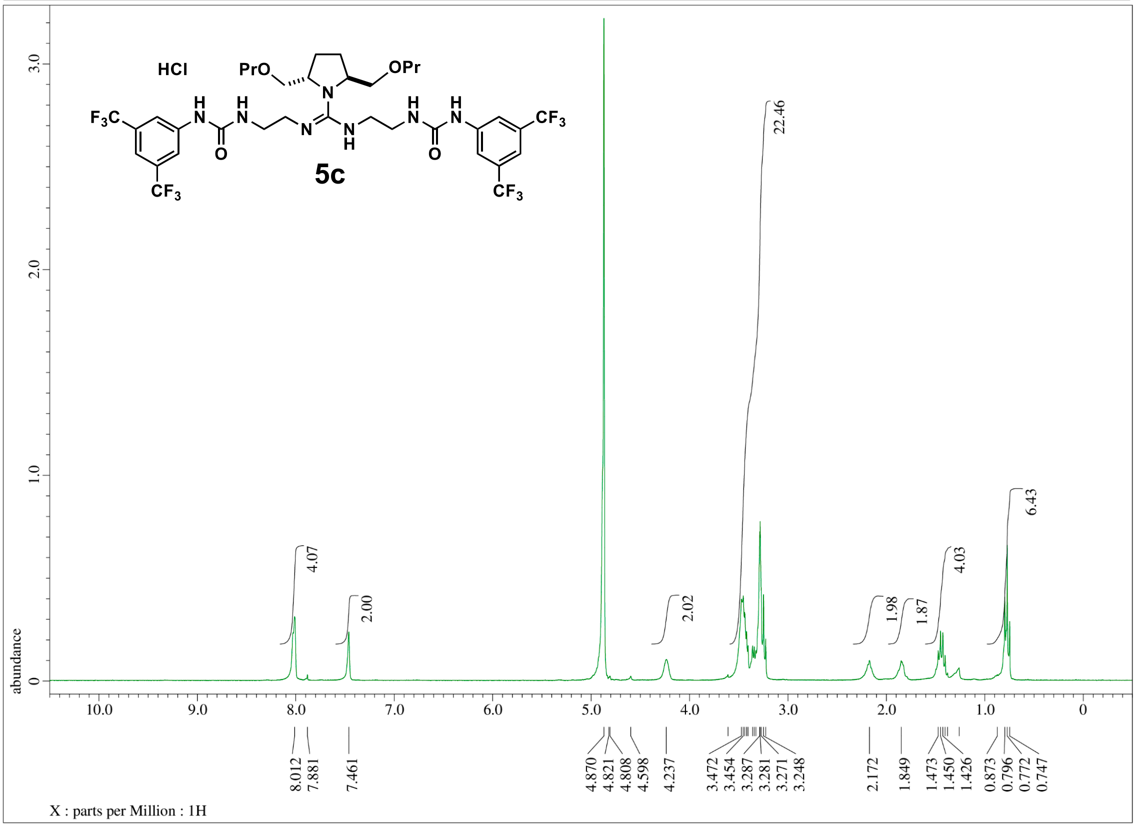

Figure S3. Copies of NMR spectra of **5c**.

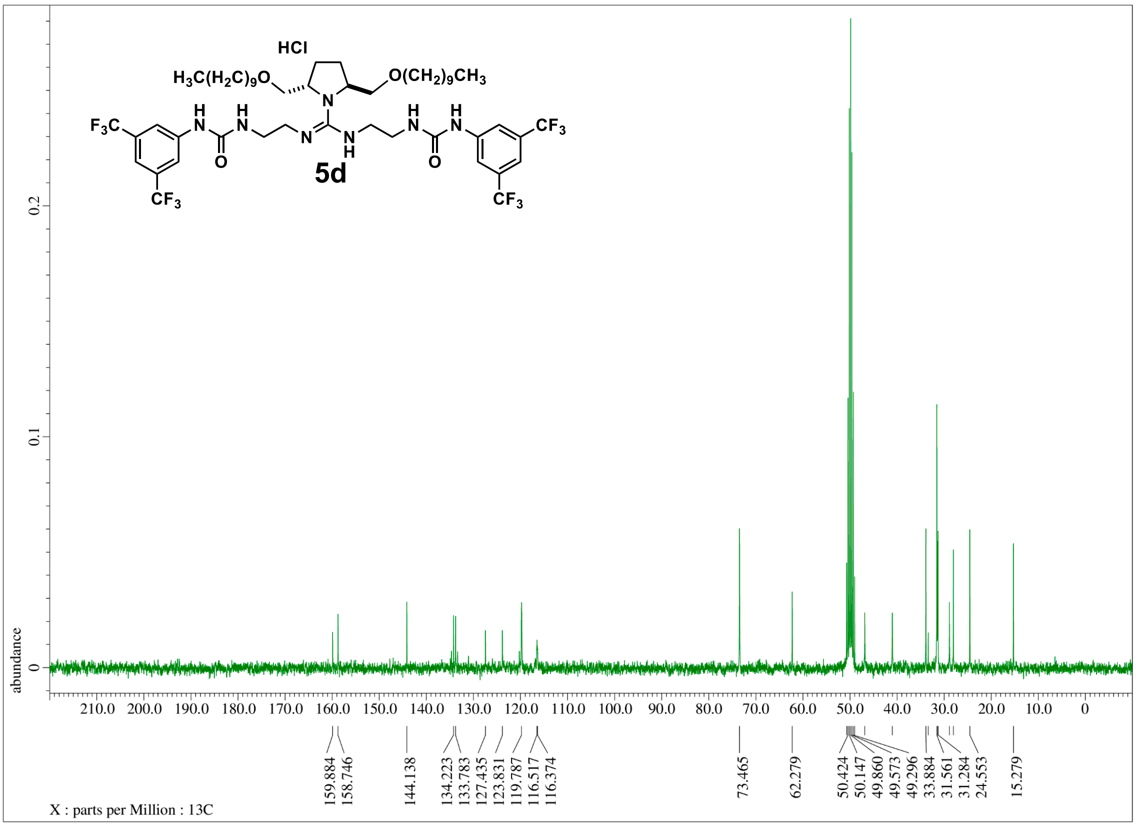

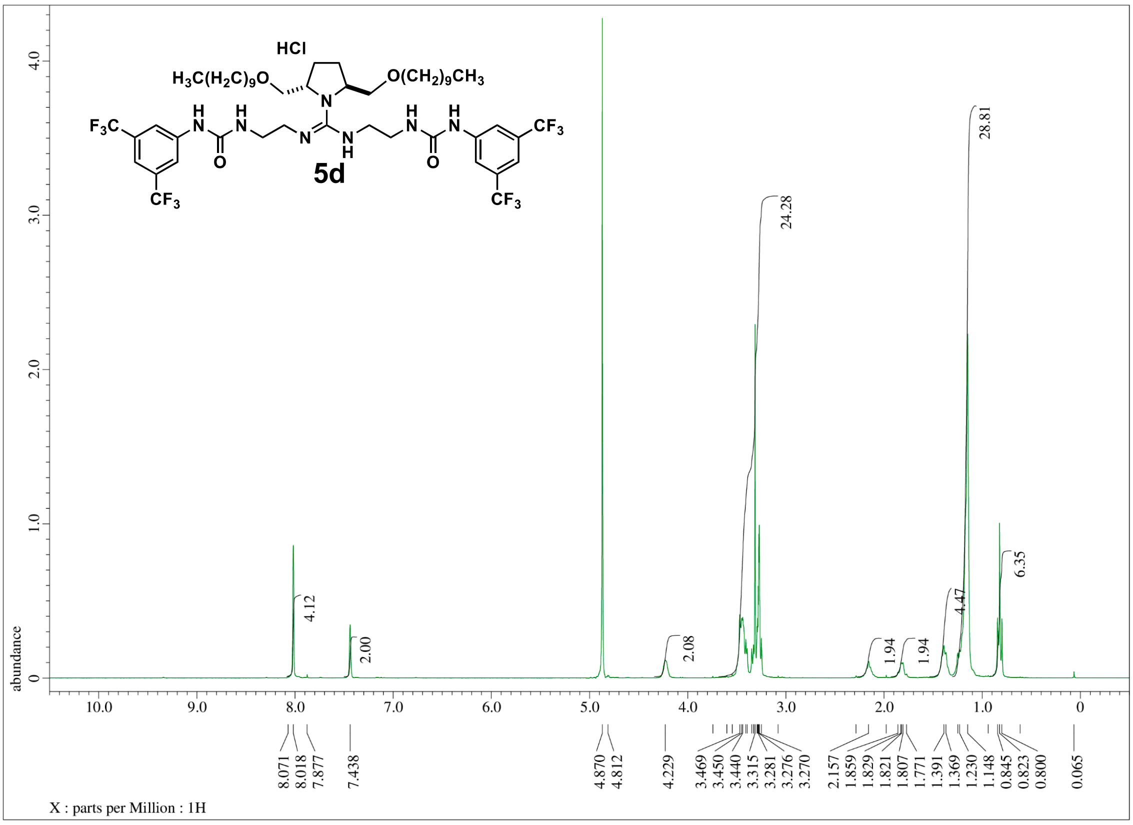

Figure S4. Copies of NMR spectra of **5d**.

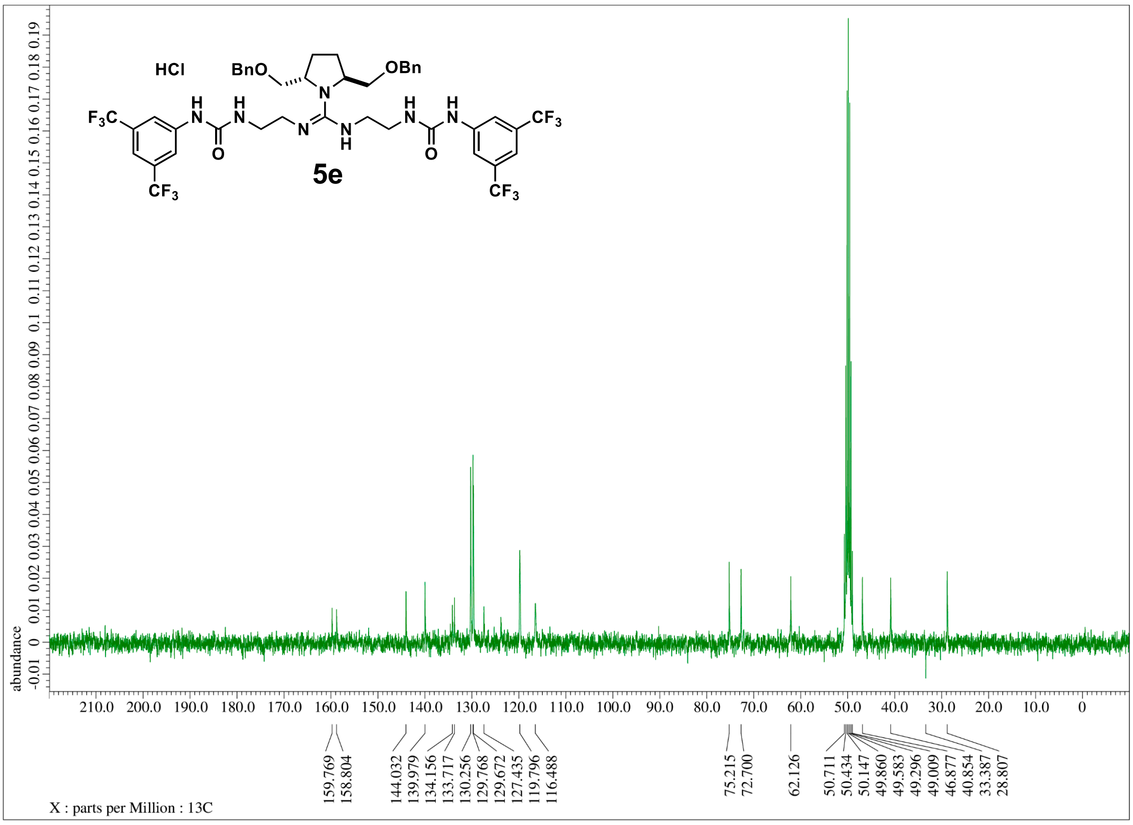

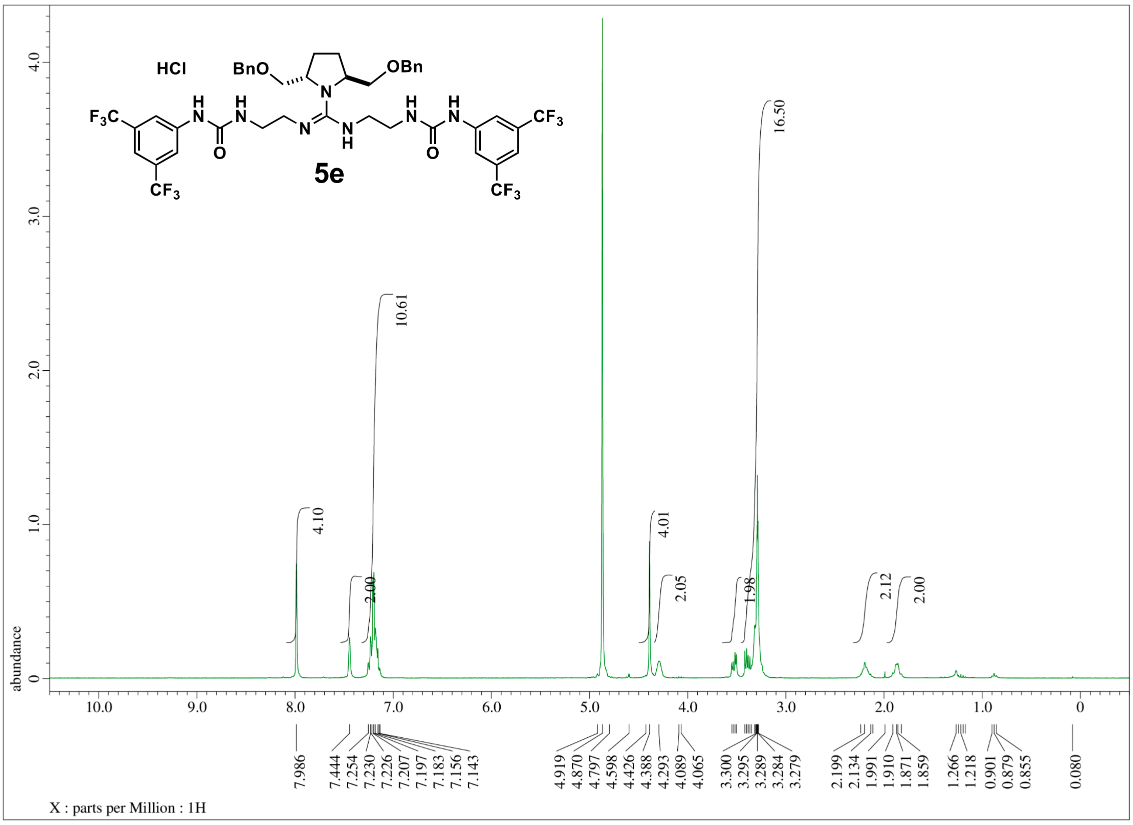

Figure S5. Copies of NMR spectra of **5e**.

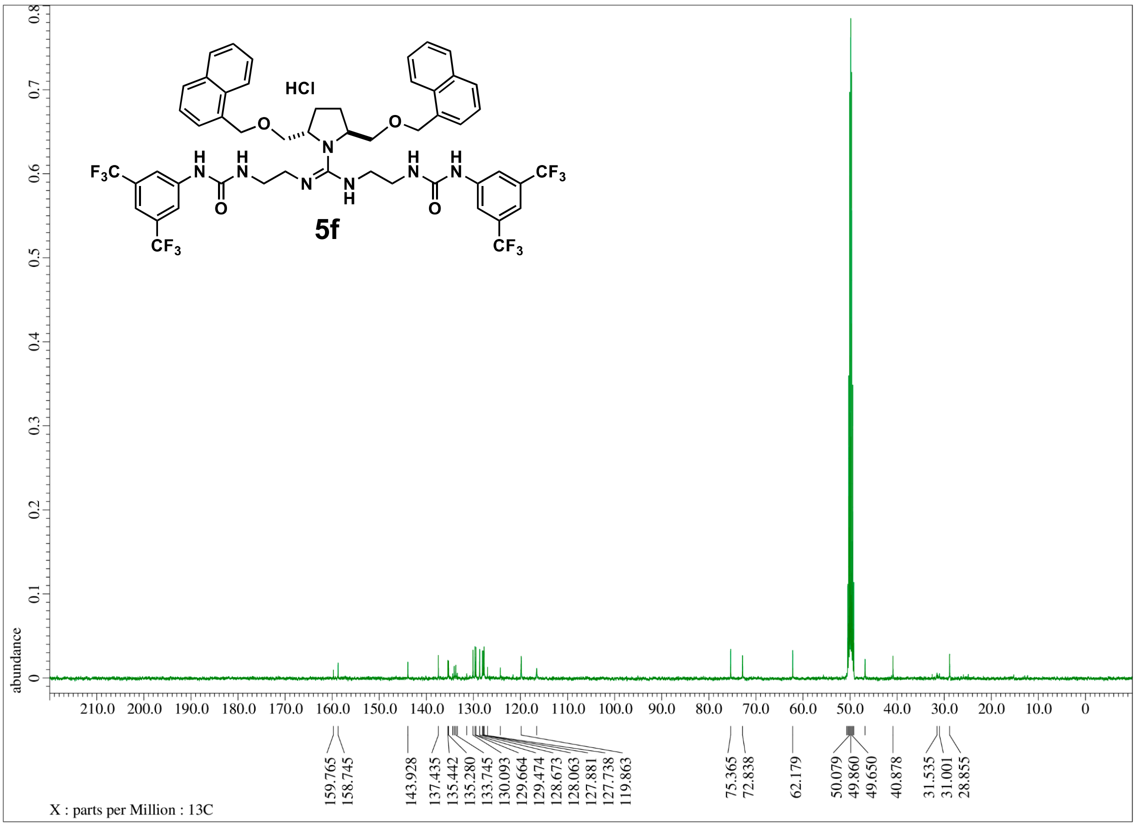

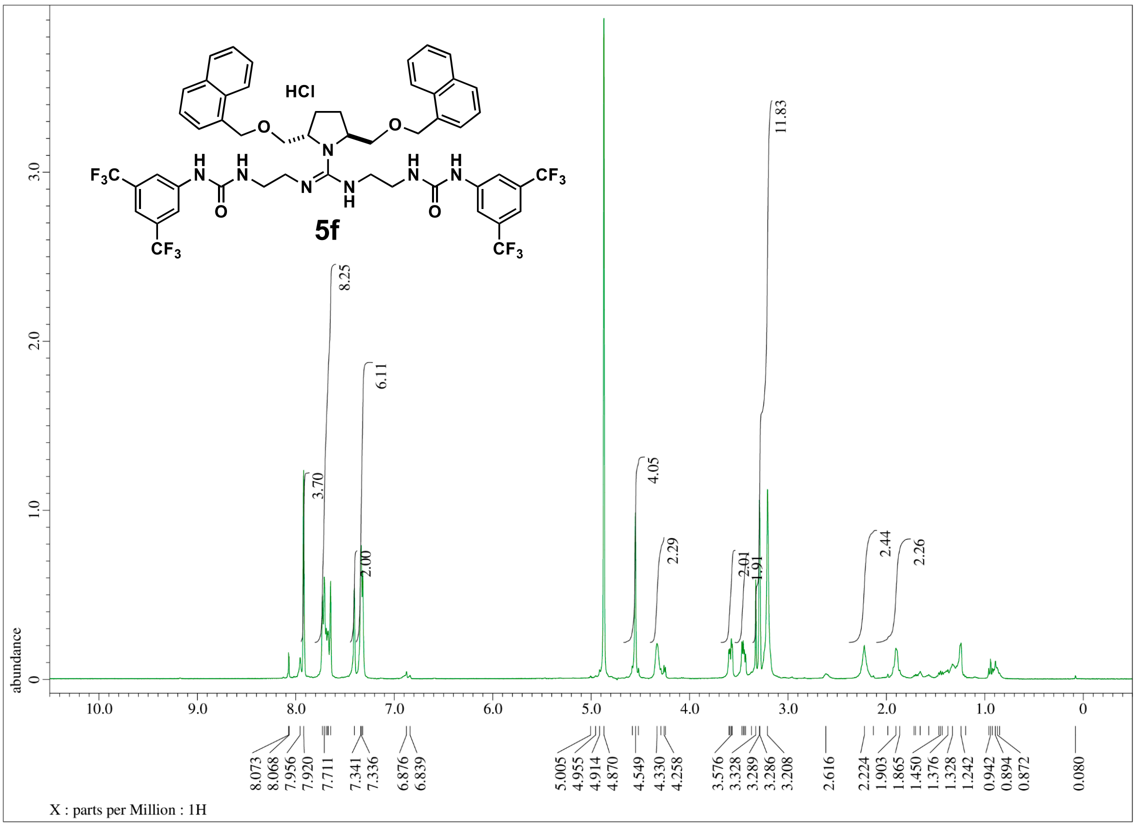

Figure S6. Copies of NMR spectra of **5f**.

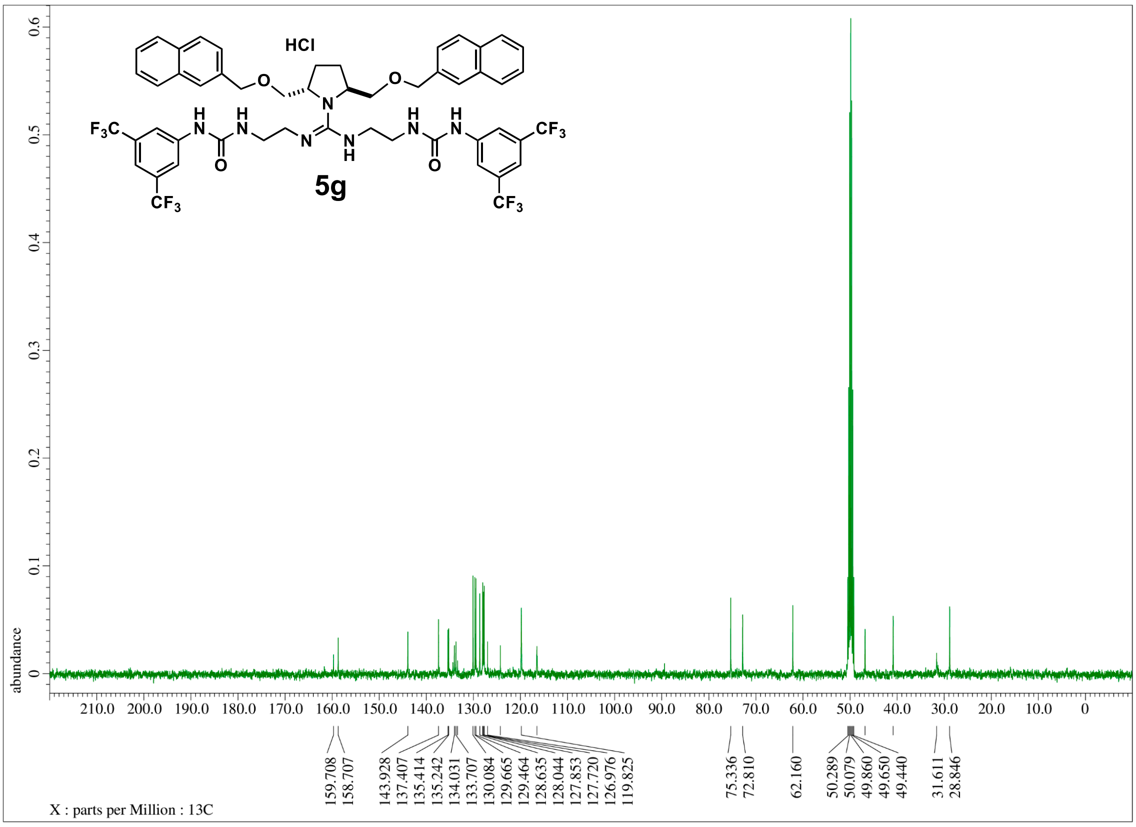

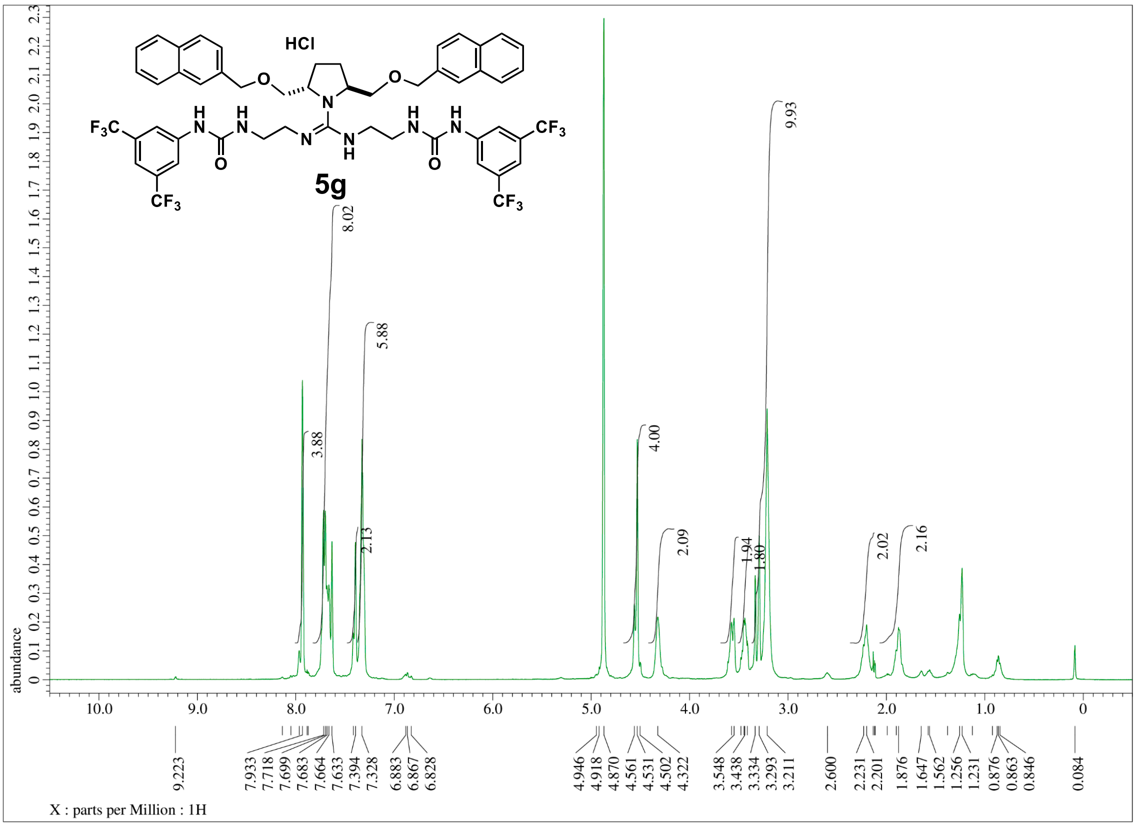

Figure S7. Copies of NMR spectra of **5g**.

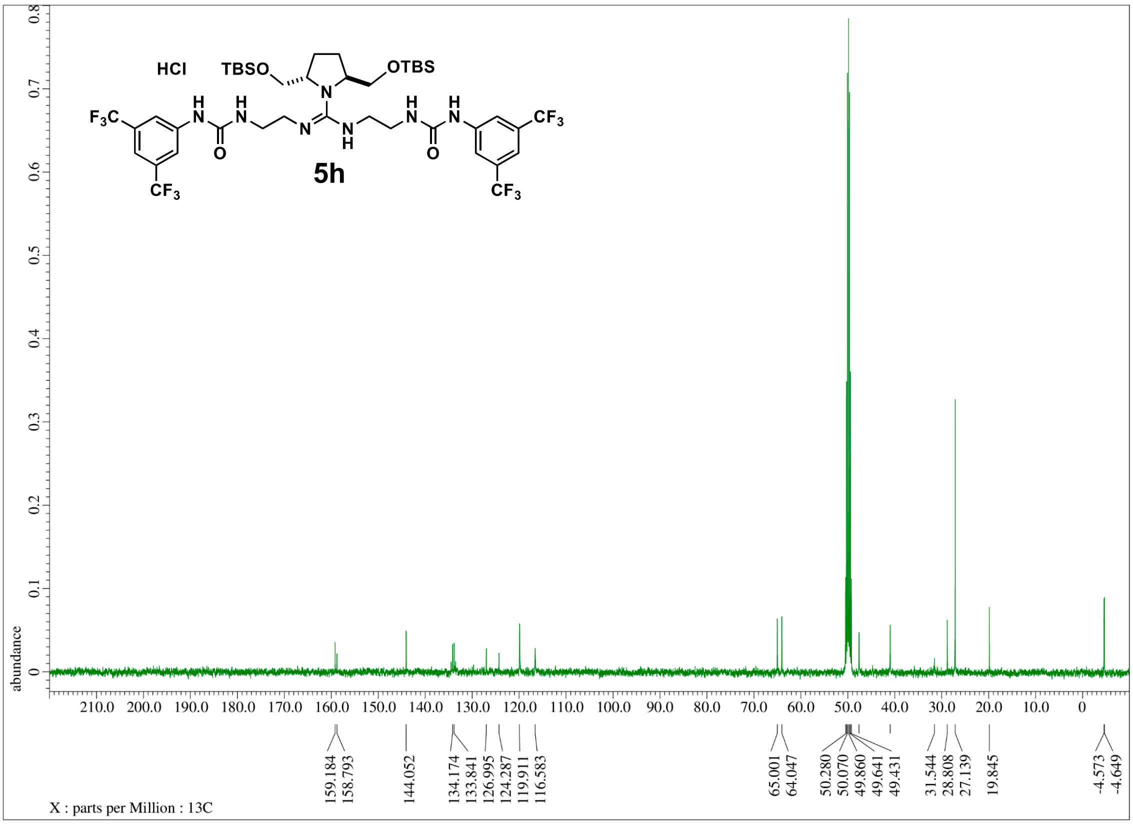

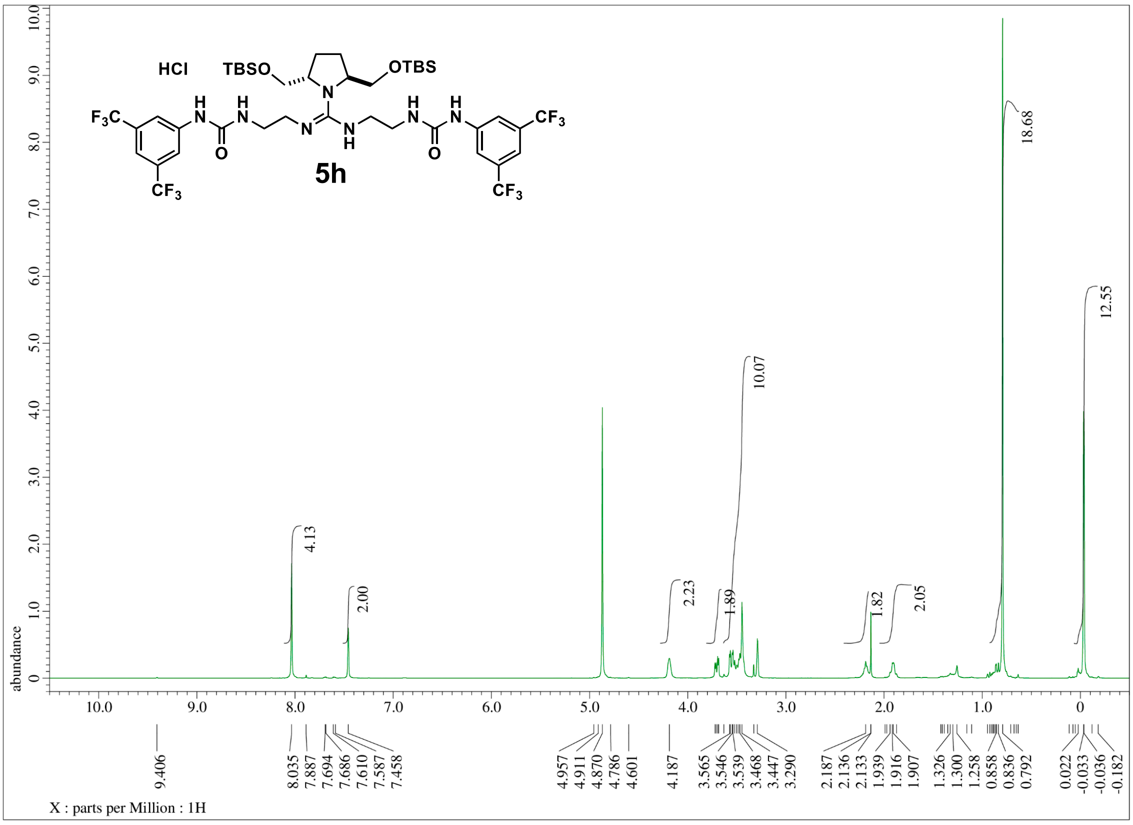

Figure S8. Copies of NMR spectra of **5h**.

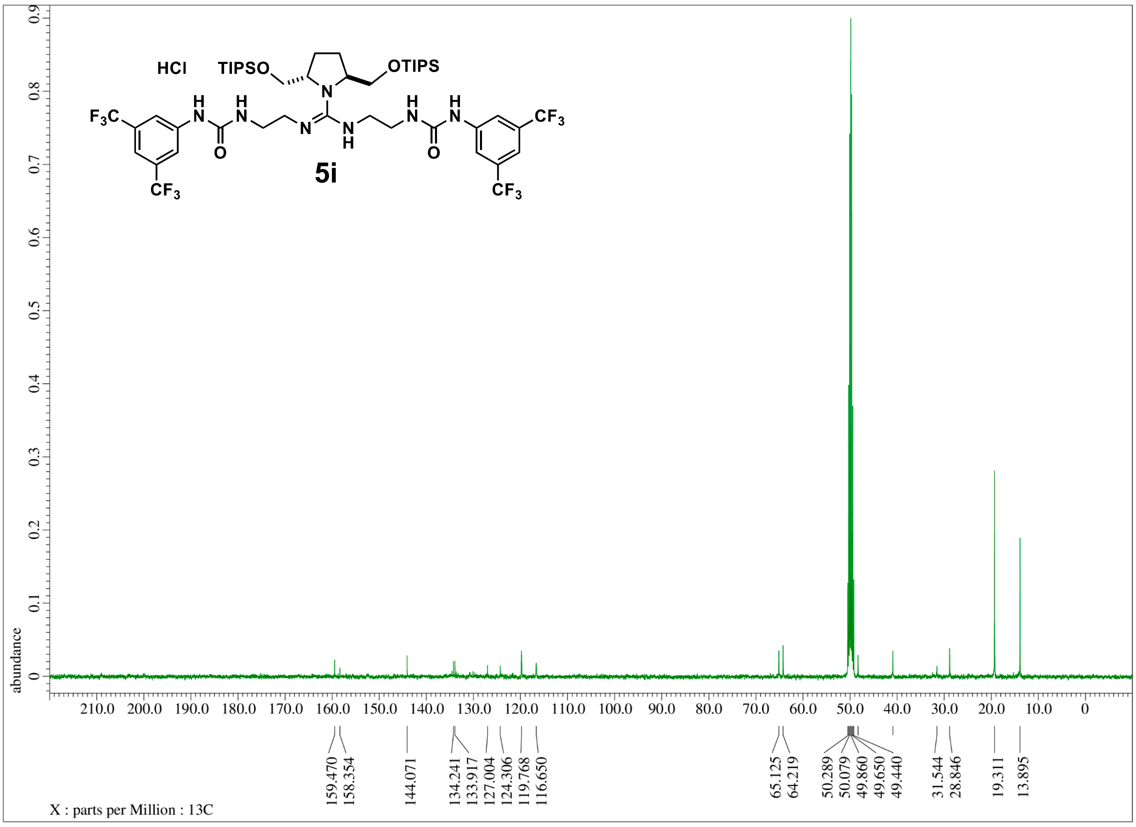

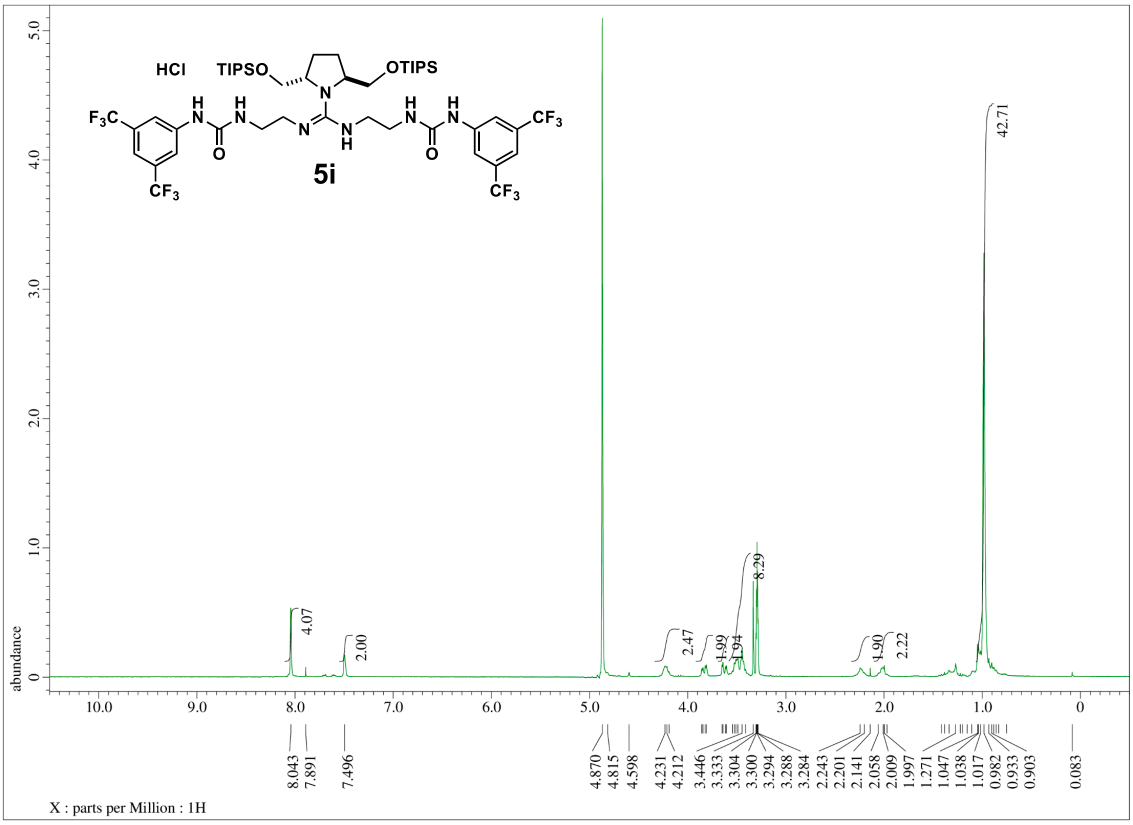

Figure S9. Copies of NMR spectra of **5i**.

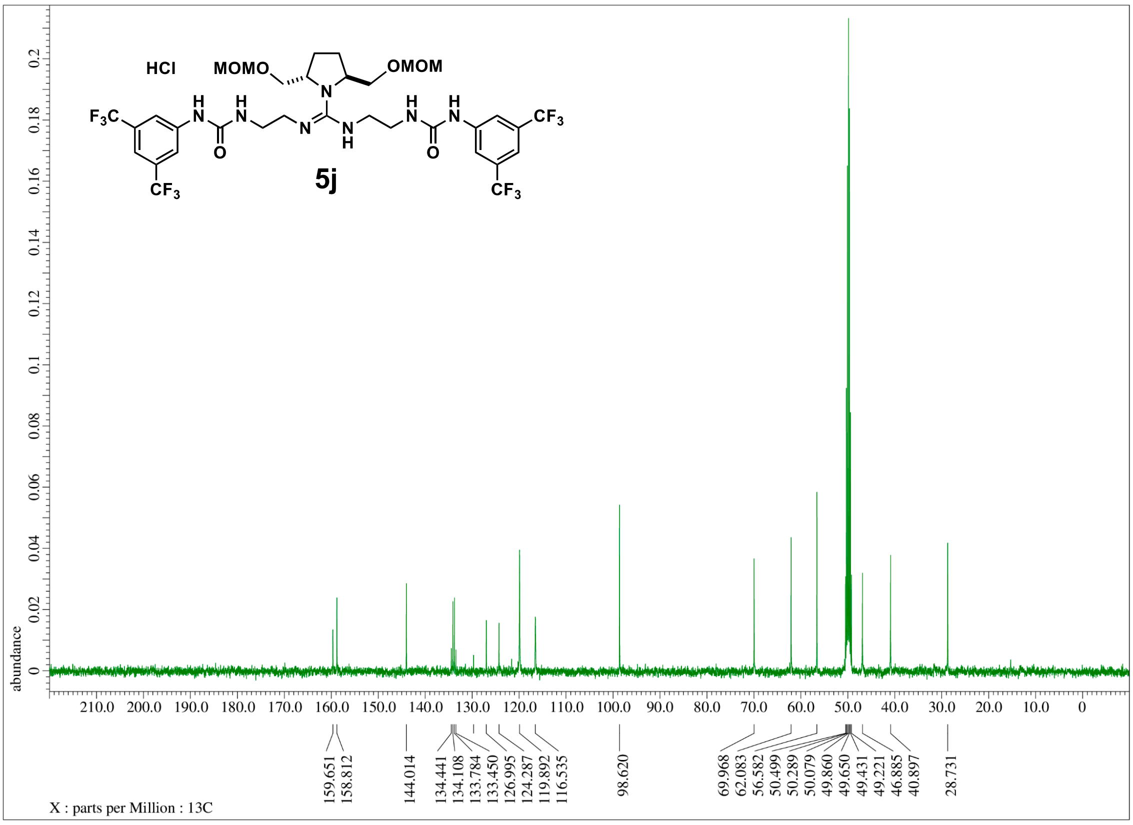

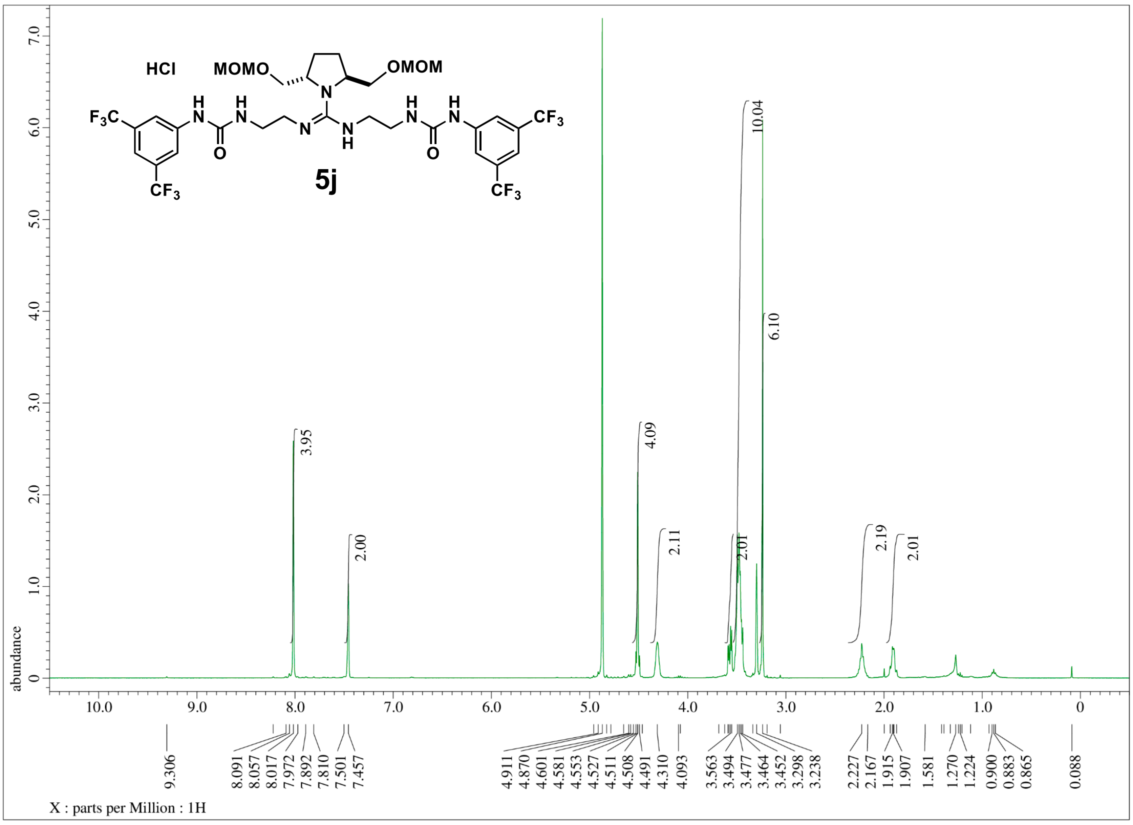

Figure S10. Copies of NMR spectra of **5j**.
